# Nanoscale quantitative surface roughness measurement of articular cartilage using second-order statistical-based biospeckle

**DOI:** 10.1371/journal.pone.0246395

**Published:** 2021-01-29

**Authors:** Doaa Youssef, Salah Hassab-Elnaby, Hatem El-Ghandoor

**Affiliations:** 1 Department of Engineering Applications of Laser, National Institute of Laser Enhanced Science, Cairo University, Giza, Egypt; 2 Faculty of Science, Department of Physics, Ain Shams University, Cairo, Egypt; Taipei Medical University, TAIWAN

## Abstract

Quantitative measurement of nanoscale surface roughness of articular cartilage tissue is significant to assess the surface topography for early treatment of osteoarthritis, the most common joint disease worldwide. Since it was not established by clinical diagnostic tools, the current studies have been suggesting the use of alternative diagnostic tools using pre-clinical methods. This study aims to measure the nanoscale surface roughness of articular cartilage tissue utilizing biospeckle which is used as a non-destructive and non-contact optical imaging technique. An experimental setup was implemented to capture biospeckle images from twelve cross-section areas of articular cartilage tissue gathered from bovine knee joints at 632 nm wavelength laser radiation. Then, to analyze the biospeckle image, a second-order statistical-based method was proposed through the combination of 308 highly correlated statistical features extracted from implemented gray-level co-occurrence matrices by employing principal component analysis. The result indicated that the measurement of the nanoscale surface roughness based on the first principal component only is able to provide accurate and precise quantitative measurement of early signs of articular cartilage degeneration up to 2500 nm.

## Introduction

Articular cartilage is a thin layer glassy-like connective tissue that covers the ends of joint bones with no nerves or blood vessels [1]. Its thickness varies according to the species, location, age, gender, and weight where it ranges from 1 mm to 6 mm in the human knee joint [2]. The articular cartilage reduces the friction between the interacting bones to permit the movements of one bone against another, supplies shock-absorbent of body and protects the bones from excessive loads where it can bear 2.5–5 times body weight during walk [3–5]. Besides, the articular cartilage is highly deformable; it can adapt to different types of loads by changing its reaction according to the type of load [2]. The structure of the articular cartilage is extremely organized which is divided into four layers according to the distance from its surface. The superficial layer is the thinnest and the outermost one that is surrounded by synovial fluid, a very high-water content (~98.8%), to lubricate the two articular cartilage surfaces in the joints capsule [3]. Healthy articular cartilage consists of a small number of cells called chondrocytes (1–5%), that are embedded in the extracellular matrix (contains water (65–80%), proteoglycans, glycoproteins, and lipids), and type II collagen [4,6]. Typically, the water is concentrated near the articular cartilage surface and decreases with increasing the depth towards the innermost layer [3]. This large amount of water helps in gas, nutrient, and waste exchange with the surrounding synovial fluid. Additionally, nearly 70% of water moves outside the articular cartilage when exposed to load for the deformation of articular cartilage helping in reducing friction. The type II collagen assists in the attachment of the chondrocytes to the extracellular matrix and maintains the tensile strength [7].

Osteoarthritis is a public health issue that describes articular cartilage degeneration [8,9]. It can be caused by the erosion of the articular cartilage tissue resulting in cracking and thinning of this connective tissue surface that in advanced stages can be gradually worn out to bone surface, i.e. naked of articular cartilage [4]. Articular cartilage of knee joints can be damaged by genetics, overweight especially in women, leg curvatures, age, knee injuries, and repeated stress on the knee like climbing stairs, cycling, and long time sitting [4,7,10]. Thus, the nanoscale average surface roughness of articular cartilage is an important symptom of osteoarthritis for the reason that the earliest sign of osteoarthritis is fibrillation of the articular cartilage surface; it has to be measured quantitatively. Yet, the clinical diagnostic tools, such as magnetic resonance imaging, computer tomography, arthroscopy, and plain x-ray provide a qualitative investigation of osteoarthritis and cannot detect osteoarthritis in the early stage [2,4,11]. Therefore, pre-clinical methods have appeared whereas they play a crucial role in investigating the integrity of articular cartilage tissue surface. A small size mechanical indentation instrument was developed to quantitatively measure the stiffness of articular cartilage during arthroscopy [12]. The authors tested the instrument in cadaver articular cartilage specimens. Moreover, for measuring the dynamic indentation properties of articular cartilage, a mechanical indentation tool was developed [13]. Whereas it was supposed that, with further development, the system could be inserted into a human or animal knee joint under arthroscopic control. However, this technique depends on imposing a constant deformation on the articular cartilage surface and the maximum indenter by which the articular cartilage resists the induced deformation was measured and used as an indicator of articular cartilage stiffness [14]. To overcome the limitation of mechanical indentation, ultrasound indentation measurements have been developed [15–18]. In these studies, the ultrasound technique was shown to be sensitive for the direct measurement of the average surface roughness of articular cartilage. The main drawback of this technique is that it requires an invasive approach in clinical use. with non-invasive ultrasound imaging, the ultrasound penetration would then be limited to small areas in the tissue. Optical coherence tomography was first introduced for the assessment of articular cartilage microstructure by Herrmann et al. [19]. The main limitation of articular cartilage OCT imaging is that the penetration of light in the cartilage is limited [20]. Several publications for measuring the articular cartilage surface ex-vivo through contact profilometers like stylus profilometer [21,22], and scanning probe microscopes [23–26] have appeared. They allowed quantitative measurement of the average surface roughness, while they can easily scratch the tissue surface, as they depend on passing a probe across the specimen surface. Besides, they allow a very small scanning area at one time (<100 μm^2^). The non-contact profilometers such as the scanning electron microscope were utilized to assess the articular cartilage surface topography [22,27]. The most disadvantages of SEM are that the specimens have to be covered with a thin layer of gold or carbon and the specimens have to be dehydrated. Hence, SEM cannot be used to scan the biological specimens in their natural conditions. Other non-contact profilometers such as scanning white light interferometer [28] and optical profilometer [29] were used to qualitatively investigate the articular cartilage surface. To the best of our knowledge, the reported values of average surface roughness for healthy and degenerated articular cartilage were varied in the existing studies according to the accuracy of the applied measurement method. For example, when utilizing the ultrasound indentation [15], the average surface roughness values were 7.9, 29.1, and 49.1 μm for smooth, intermediate, and rough articular cartilage surfaces, respectively. In another report utilizing the optical profilometer [29], the average roughness of healthy and degenerated surfaces was reported to be 30 ± 5 and 140 ± 9 μm, respectively. While, the scanning white light interferometer [28] gave values of 800 ± 300, 1000 ± 300 and 1700 ± 900 nm for the average roughness of osteoarthritis gardes 0 (healthy), 1, and 2, respectively. Examination using scanning probe microscopes, like atomic force microscope (AFM), was reported to be 68.90, 110.40, 110.95, and 119.22 nm for grades 0, 1, 2, and 3, respectively [26].

Speckle is observed in any imaging modality involving laser illumination such as optical coherence tomography and ultrasound. Therefore, the speckle is treated as noise that distorts the results of measurements and, subsequently, has to be eliminated [30]. Though, speckle image contains significant information about the observed object surface; that is why speckle imaging methods have been of interest to researchers [30,31]. Since then, through speckle imaging, never-ending studies in many fields of medicine, engineering, food quality assessment, agriculture, industry, and science have been developed [32–34]. It is worth noting that speckle is popularly called biospeckle when characterizing a biological tissue [32]. Regarding the biological tissue, several reports on laser speckle rheology (LSR), which measures the biomechanical properties of tissues and biofluids, have been provided [35–39]. Another possible utilization of biospeckle is laser speckle flowgraphy, which is a powerful tool for blood flow mapping [40–42]. A simple algorithm based on laser speckle contrast imaging (LSCI) and histogram analysis of biospeckle data to study the cerebral blood flow in rat cortex was described [43]. Surface configuration for the normal and laser-treated retina to study the accumulation effects on the retina was investigated through laser photography [44]. The surface roughness variations of zirconia materials that were used for dental crowns were measured by speckle-based autocorrelation analysis [45]. Deana et al. [46] reported on the application of biospeckle imaging for the early assessment of carious lesions on teeth.

**Fig 1:**
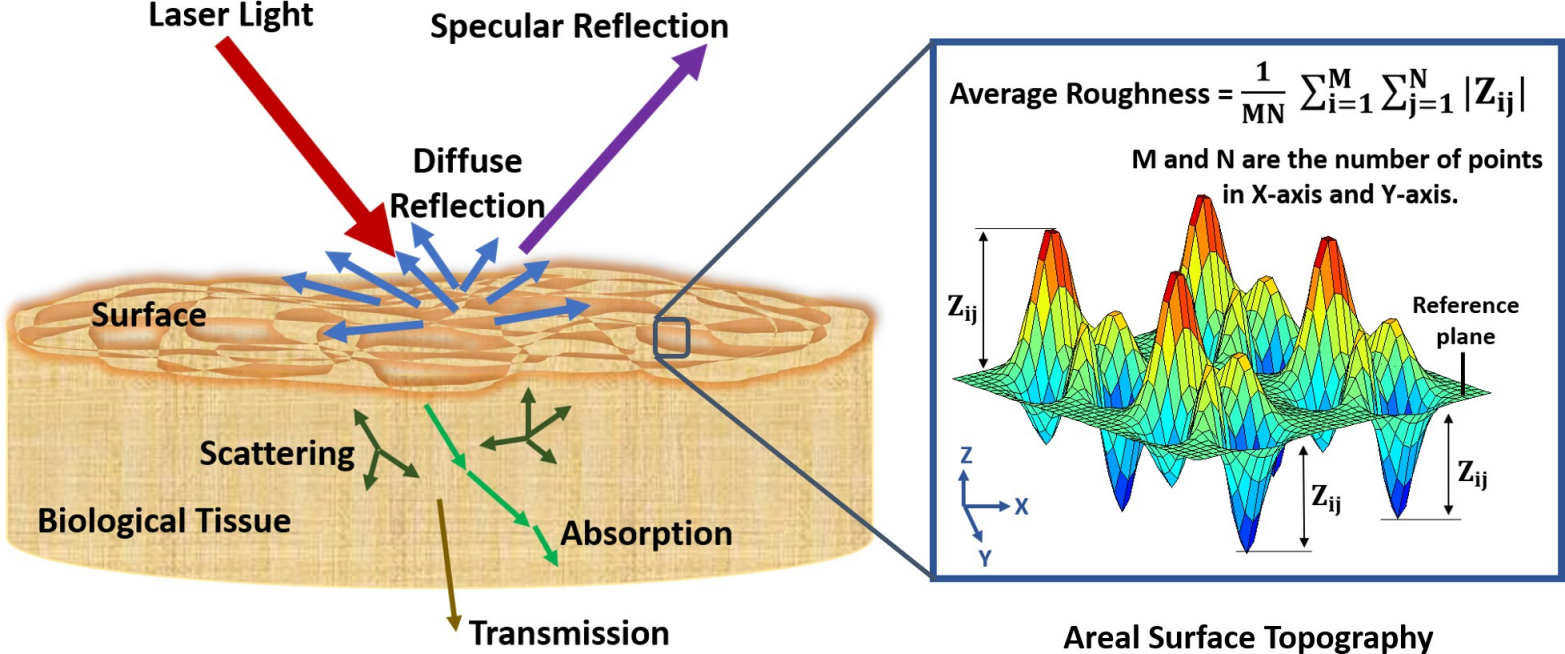
Shematic presentation of laser interaction with biological tissue.

Generally, when laser light illuminates a biological tissue as presented in Fig 1, some effects may exist: reflection (specular and/or diffuse), scattering, transmission, and/or absorption [47,48]. The biospeckle imaging is based on the backscattered light, due to diffuse reflection, from the biological tissue surface [49]. Where the different points on the biological tissue surface transmit spherical wavelets that are subjected to different path lengths due to the surface topography (see Fig 1). As all the transmitted spherical wavelets are coherent, they interfere with each other resulting in an illuminated chaotic and irregular pattern with bright and dark patches covering the biological tissue surface termed biospeckle image [31]. The bright and dark patches, respectively, correspond to constructive and destructive interference [31,50]. Thus, the biospeckle image is characterized by random intensities and phases distribution [51].

In consequence of the texture properties of the biospeckle image, speckle contrast technique based on first-order statistics to characterize surface roughness was used by Persson [52]. Dhanasekar et al. [53] extracted autocorrelation parameters from the speckle images to investigate the surface roughness. Besides, some studies binarized the speckle images and their statistical properties were utilized to evaluate the surface roughness [54–57]. By employing the gray-level co-occurrence matrix of the speckle image, the authors could measure the surface roughness [58–60]. A local contrast analysis model was proposed to extract three parameters from the biospeckle images for the evaluation of the surface roughness [11]. By Hurst exponent method, Peron et al. [30], Sampaio et al. [61], and Martinez et al. [62] analyzed the speckle images. Moreover, local texture analysis through morphological operations by opening and closing at different neighborhoods was applied to investigate the speckle images [63].

The proposed method is based on the statistics of the gray-level co-occurrence matrix (two-dimensional histogram) and principal component analysis for investigating the biospeckle images to quantitively measure the nanoscale average surface roughness of articular cartilage tissue specimens gathered from bovine knee joints.

## Speckle theory

**Fig 2:**
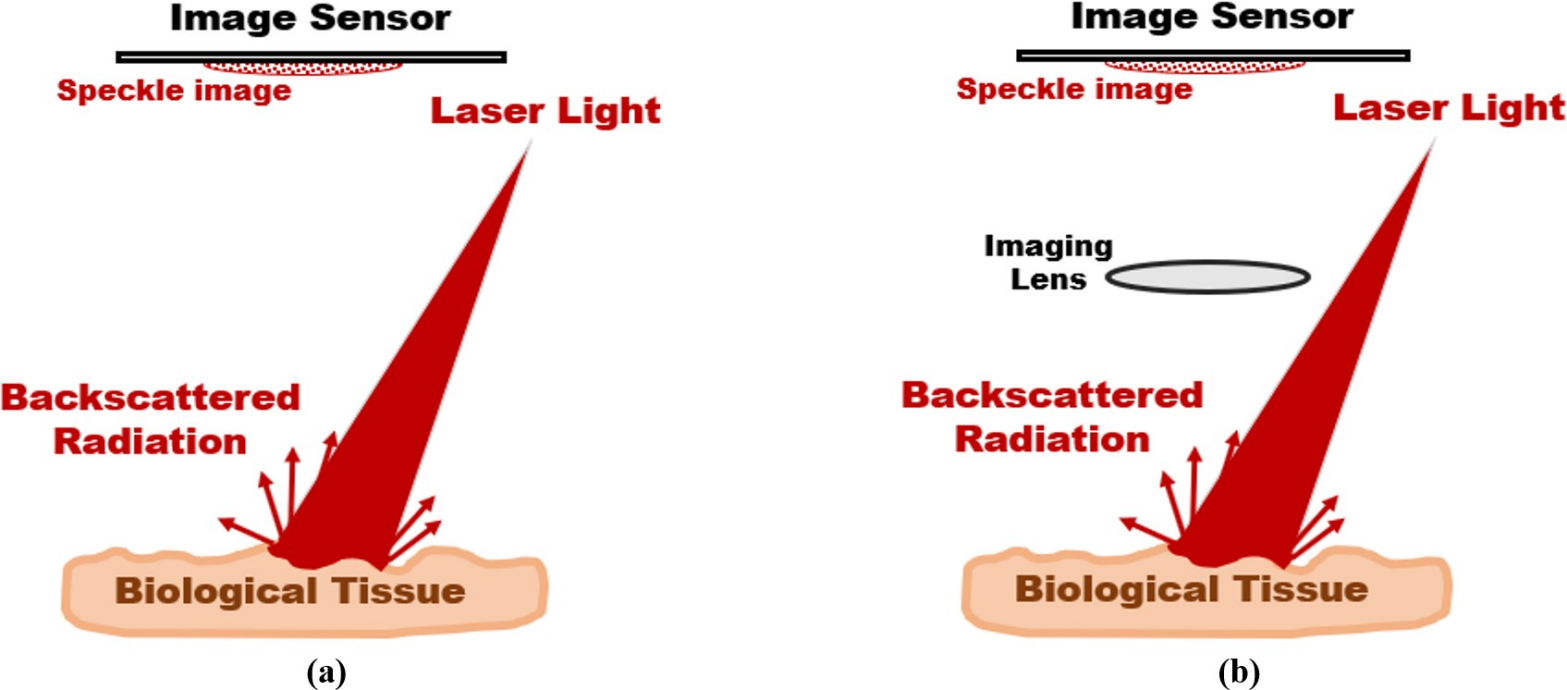
Two-basic biospeckle imaging configurations for collecting biospeckle images of a biological tissue surface: (a) objective speckle and (b) subjective speckle.

The two-basic configurations for recording biospeckle images to investigate the surface roughness of a biological tissue surface are shown in Fig 2. In Fig 2(A), the biospeckle image is formed in free-space and termed objective speckle or speckle at the diffraction plane. The average size of the speckle grains at the observation plane is approximately given by [64]:

$$\delta_{O}=1.22\;\lambda \;L/D$$ (1)

where λ is the wavelength of the laser radiation, L is the distance from the rough surface to the observation plane and D is the diameter of the illuminated spot. This equation shows that when the diameter of the illuminated spot is wider, the speckle is smaller.

While as shown in Fig 2(B), the biospeckle image that is formed on the image plane of an imaging lens is termed subjective speckle [31]. In this case, the average speckle grain size is approximately defined as follows [64]:

$$\delta_{S}=1.22\;\lambda (1+M)f/d$$ (2)

where, f, M, and d are the focal length, the magnification, and the diameter of the imaging length, respectively. From Eq (2), it can be concluded that when the aperture of the imaging lens is wider, the laser speckle is smaller. This is to be expected, as the size of the diffraction pattern of a lens decreases as the aperture of the lens increases [65]. Hence, under the same experimental conditions, the texture properties of the biospeckle image will depend only on the topography of the biological tissue surface. In this study, the biospeckle images were recorded through the objective speckle.

## Experimental configuration

### Specimen source and preparation

Ex-vivo study on bovine articular cartilage on bone specimens was utilized in this study. They were directly collected after the slaughter at a local butcher’s shop (Behiry Butcher, Giza, Egypt). For documented results, three specimens, approximately 2 cm ×2 cm × 2cm inspected with the naked eye to ensure that they are free from damage, were obtained from the lateral and medial tibial condyle of a male bovine articular capsule of the right knee joint, approximately three years old. Then, four cross-section areas on each specimen surface were degenerated using an abrasive machine into different average roughness, R_a_, values. Finally, before the imaging process, the average roughness of the twelve degenerated cross-section areas of the specimens’ surfaces was measured using a stylus profilometer. Notably, the specimens were transported in an icebox and immediately disposed after recording the biospeckle images.

### Optical setup

**Fig 3:**
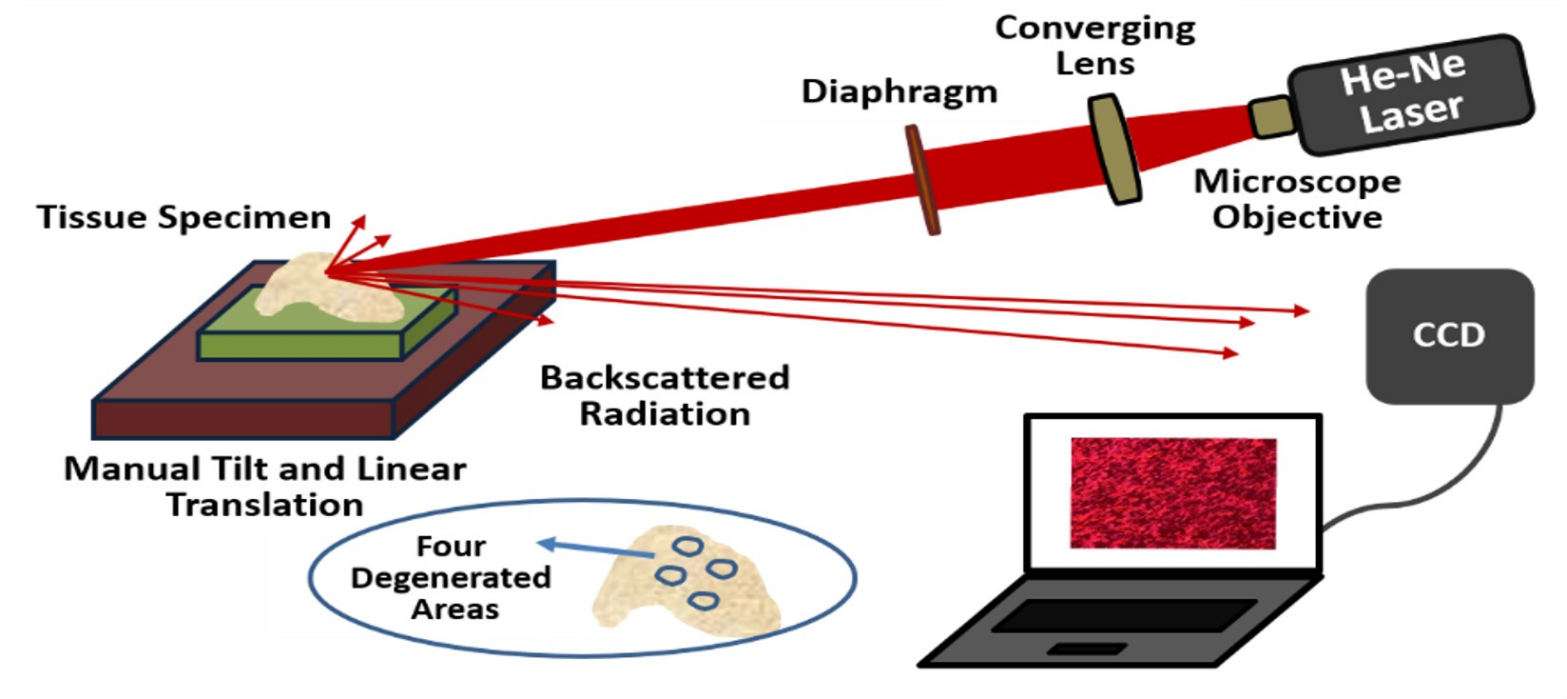
Schematic presentation of the biospeckle optical setup.

As the scattering properties of the articular cartilage tissue dominates its absorption properties in the wavelength range ~ 400–850 nm [58,66], the optical setup was built with 5 mW, 632 nm He-Ne laser. The laser beam was then expanded to 4 mm in diameter after a diaphragm to provide local investigations of the specimen which was placed on manual tilt and linear translation stages to be examined for the different degenerated areas. The biospeckle images produced from the interference of backscattered radiations were recorded with a CCD camera of resolution 4608×3456 pixels that were placed at 50 cm from the specimen (Fig 3). During the biospeckle imaging, all the experimental conditions and the angle between incident laser radiation and the normal direction (~ 15˚) were respected.

## Biospeckle statistical-based image analysis

### Statistics of gray-level co-occurrence matrix

The gray-level co-occurrence matrix (GLCM), innovated by Haralick et al. [67], is a two-dimensional histogram of size L×L, where L is the maximum gray-level value of an image. The GLCM is a second-order statistical-based analysis since it is implemented from the texture information of the image by calculating the frequency of occurrence of two-neighbor pixels of values i and j, respectively, separated by a distance d with a specific direction θ. Generally, symmetrical GLCM around its principal diagonal (i.e. in the direction of i = j) is implemented when the calculation is done in the forward and backward along four directions corresponding to angles of 0° (horizontal), 45° (right diagonal), 90° (vertical) and 135° (left diagonal). Assuming a biospeckle image Z having a dimension of M×N, the values contained in the cells (i,j) of the GLCM, implemented from Z, is defined by the following equation [67]:

$${\mathrm{CM}(i,j)}_{d,\theta }=\sum_{x=1}^{M}\sum_{y=1}^{N}\{\begin{array}{l} 1,\;if\;Z(x,y)=i\;and\;Z(x+d,y+d)=j\\ 0,\;otherwise \end{array}$$ (3)

To avoid scaling effects, it is preferred to use normalized values of CM instead of frequency. The normalized CM, denoted by P, is given by Eq (4) [67]. Thus, the sum of all cell’ values of P is equal to one.

$$P(i,j)=\frac{\mathrm{CM}(i,j)}{\sum_{i=0}^{L‐1}\sum_{j=0}^{L‐1}\mathrm{CM}(i,j)}$$ (4)

In view of the GLCM, it can measure the uniformity or the randomness of the biospeckle image. For example, when comparing two images with few and many gray-level transitions, the more uniform one (few transitions) assigns the GLCM cells with high values within the principal diagonal region. While the principal diagonal region of the other image (many transitions) is modified by low values. Therefore, the GLCM elements’ distribution (spatial variation) and values depend on the texture properties of the image. To describe the GLCM, Haralick et al. [67] extracted 14 statistical parameters from the GLCM, for each direction and separation distance. This study deals with eight of Haralick’s statistical parameters which describe the image homogeneity, contrast, randomness, and gray-level transitions. Their mathematical expressions and descriptions are listed in Table 1.

**Table 1:** Representation and description of Haralick’s statistical parameters.

Statistical parameter	Mathematical expression [67]	Description
**Angular second momentum (ASM)**	$\mathrm{ASM}=\sum_{i=0}^{L‐1}\sum_{j=0}^{L‐1}P^{2}(i,j)$	It measures the image homogeneity. A high value of this statistical parameter is obtained for a homogeneous image which is characterized by very few gray-level transitions and hence the matrix P is modified by few entries to the GLCM cells with large values.
**Contrast (CON)**	$\begin{array}{c} CON=\sum_{n=0}^{L-1}n^{2}\left\{\sum_{i=0}^{L-1}\sum_{j=0}^{L-1}P(i,j)\right\}\\ |i-j|=n \end{array}$	It is a measure of the local variations of the gray-level intensities within an image whereas it gives weight factors, denoted by n^2^, to the matrix P with respect to the distances away from the GLCM principal diagonal. The principal diagonal is assigned by n^2^ = 0, the adjacent diagonals above and below the principal diagonal are assigned by n^2^ = 1, etc. A high value of this measure is obtained when the elements accumulate away from the GLCM principal diagonal.
**Inverse difference moment (IDM):**	$\mathrm{IDM}=\sum_{i=0}^{L‐1}\sum_{j=0}^{L‐1}\frac{1}{1+{\left(i‐j\right)}^{2}}P(i,j)$	It is a measure of the closeness of the GLCM cells to the principal diagonal by modifying the matrix P with a weight factor 1/1+(i—j)^2^.
**Entropy (ENT):**	$\mathrm{ENT}=-\sum_{i=0}^{L‐1}\sum_{j=0}^{L‐1}P(i,j)\;\log\;P(i,j)$	It measures image disorder and randomness. It outputs a higher value for an image with high gray-level transitions, i.e. modifies the matrix P with small values to many cells.
**Difference average (DA)**	$\mathrm{DA}=\sum_{i=0}^{L‐1}iP_{x‐y}(i)$	$\begin{array}{c} P_{x‐y}(k)=\sum_{i=0}^{L‐1}\sum_{j=0}^{L‐1}P(i,j),\;k=0,1,2,\ldots \ldots ,L‐1\\ |i‐j|=k \end{array}$ The value at P_x-y_(0) is the summation of elements accumulated in the principal diagonal and the value at P_x-y_(k) is the summation of the elements in the k^th^ diagonal above and below the principal diagonal. Like CON and ENT, high values of DA, DV, and DE are obtained when the elements distribute away from the principal diagonal.
**Difference variance (DV)**	$\mathrm{DV}=\sum_{i=0}^{L‐1}{\left(i-DA\right)}^{2}P_{x‐y}(i)$	
**Difference entropy (DE)**	$\mathrm{DE}=-\sum_{j=0}^{L‐1}P_{x‐y}(i,j)\;\log\;P_{x‐y}(i,j)$	
**Correlation (CORR)**	$\mathrm{CORR}=\sum_{i=0}^{L‐1}\sum_{j=0}^{L‐1}P(i,j)\frac{\left(i‐\mu_{x})(j‐\mu_{y}\right)}{\sigma_{x}\sigma_{y}}$	$\mu_{x}=\sum_{i=0}^{L‐1}\sum_{j=0}^{L‐1}\mathrm{iP}(i,j)\;\sigma_{x}=\sqrt{\sum_{i=0}^{L‐1}\sum_{j=0}^{L‐1}{\left(i‐\mu_{x}\right)}^{2}P(i,j)}$ $\mu_{y}=\sum_{i=0}^{L‐1}\sum_{j=0}^{L‐1}\mathrm{jP}(i,j)\;\sigma_{y}=\sqrt{\sum_{i=0}^{L‐1}\sum_{j=0}^{L‐1}{\left(j‐\mu_{y}\right)}^{2}P(i,j)}$ where μ_x_, μ_y_ and σ_x_, σ_y_ are the means and standard deviations of rows and columns, respectively. This statistical parameter measures the correlation between cells in the rows and columns of P.

### Principal component analysis

Unfortunately, visualizing the relationships of more than three statistical features is extremely difficult. Therefore, it is more useful to reduce the number of statistical features under consideration by generating a new feature vector, this process is known as dimensionality reduction from variables. Then, dimensionality reduction is the linear or nonlinear transformation of the original dataset, using all statistical features, to a new dataset with a reduced number of statistical features [68].

Though many techniques have been developed for dimensionality reduction, principal component analysis (PCA) is one of the most widely used. Generally, the principal component analysis is a multivariate statistical method that generates a new set of statistical features, orthogonal to each other, called principal components, whereas each principal component is a linear combination of the original features. Since all the principal components are orthogonal axes in space, uncorrelated statistical features, there is no redundant information. If the first few principal components account for most of the variation, then only these principal components will be used to describe the data, thus leading to a dimensionality reduction [68,69]. It is important to consider that PCA is sensitive to the rating and relative scaling (i.e. dynamic range) of the statistical features. Besides, it must be applied to highly correlated statistical features (the pairwise correlation among the statistical features is more than 0.8) [68].

#### Statistical parameter preparation

Let the statistical feature dataset to be analyzed by PCA comprises n observations described by p statistical parameters and is represented by n×p data matrix X whose j^th^ column is a vector x_j_ of observations described by the j^th^ statistical parameter, that is:

$$X=\left[\begin{array}{ccccc} X_{1,1} & X_{1,2} & \ldots & \ldots & X_{1,p}\\ \vdots & \vdots & \ddots & \ldots & \vdots \\ X_{2,1} & X_{2,1} & \ldots & \ldots & X_{2,1}\\ X_{n,1} & X_{n,2} & \ldots & \ldots & X_{n,p} \end{array}\right]$$

where x_j_ = {X_1,j_, X_2,j_,…,X_n,j_}, j = 1,2,…, p.

#### Statistical parameters selection

The correlation coefficient is a statistical measure by which the strength and the direction of a relationship between two statistical parameters are measured. The range of values for the correlation coefficient is bounded between -1.0 for perfect negative correlation and 1.0 for perfect positive correlation when comparing a statistical parameter to itself. A correlation coefficient of 0.0 shows no relationship between the two statistical parameters while a correlation coefficient greater than 0.8 is generally described as strong. The correlation coefficient $r_{x_{i},x_{k}}$, where x_i_ and x_k_ are two statistical parameters vectors collected from n observations, can be computed in terms of the covariance of x_i_ and x_k_, denoted by ${\mathrm{cov}}_{x_{i},x_{k}}$, by [70]:

$$r_{x_{i},x_{k}}=\frac{{\mathrm{cov}}_{x_{i},x_{k}}}{\sigma_{x_{i}}\sigma_{x_{k}}}$$ (5)

$${\mathrm{cov}}_{x_{i},x_{k}}=\frac{1}{n‐1}\sum_{l=1}^{n}{\left(x_{i}(l)‐m_{x_{i}}\right)}^{*}(x_{k}(l)‐m_{x_{k}})$$ (6)

$$m_{x_{j}}=\frac{1}{n}\sum_{l=1}^{n}x_{j}(l)$$ (7)

$$\sigma_{x_{j}}=\sqrt{\frac{1}{n‐1}\sum_{l=1}^{n}{\left(x_{j}(l)‐m_{x_{j}}\right)}^{2}}$$ (8)

where x_j_ denotes a statistical feature vector collected from n observations, $m_{x_{i}},\;m_{x_{k}}$ and $\sigma_{x_{i}},\;\sigma_{x_{k}}$ are respectively their means and standard deviations defined by Eqs (7) and (8), respectively, and the superscript ^“*”^ denotes the complex conjugate. For the dataset X which contain more than two statistical features, the pairwise correlation matrix can be obtained directly from the dataset X using the following equation [70]:

$$R=\frac{1}{n‐1}{\left(\frac{X‐m}{\sigma }\right)}^{T}\left(\frac{X‐m}{\sigma }\right)$$ (9)

where m and σ are the mean and standard deviations vectors of the data matrix X, the superscript ^“T”^ denotes the transpose operation of a matrix.

#### Statistical feature standardization

In view of that, the extracted statistical features have been gathered with different dynamic ranges, they have to be standardized to ensure that all of them have the same dynamic range and weight before applying the PCA analysis. Consequently, each column vector in the data matrix X, i.e. a statistical feature vector x_j_, is standardized as follows [70]:

$${\bar{x}}_{j}=\frac{x_{j}‐m_{x_{j}}}{\sigma_{x_{j}}}$$ (10)

where ${\bar{x}}_{j}$ is the standardized vector of the statistical feature vector denoted by x_j_, and $m_{x_{j}}$ and $\sigma_{x_{j}}$ are the mean and standard deviation of x_j_ as defined by Eqs (7) and (8), respectively. By statistical feature standardization, the values of each statistical feature will have zero-mean and unit variance.

#### Finding the principal components

Generally, the principal components Y of the statistical feature dataset is a matrix containing the linear combination of the columns of the data matrix as follows [69]:

Y=XA (11)

where A is a matrix of weight coefficients containing a set of p eigenvectors computed from p×p covariance matrix of the dataset X, in which each column vector a_j_ = {A_1,j_, A_2,j_,…,A_p,j_} represents one eigenvector. Then, each column vector Y_j_ = {Y_1,j_, Y_2,j_,…,Y_n,j_} in the matrix Y will represent one principal component. Since the set of eigenvectors form an orthonormal set, these principal components are linearly independent and uncorrelated. As follows, the principal components are computed from the dataset X according to the following steps:

**Step 1:** an n×n covariance matrix, C, is computed from the dataset X which has zero mean by [69]:

$$C=\frac{1}{n‐1}X^{T}X$$ (12)

To the best of our knowledge, the covariance matrix C is the same as the pairwise correlation matrix R, obtained from (9), as the dataset X has unit variance and zero mean.

**Step 2:** a set of p scalar eigenvalues λ = λ_1_, λ_2_,…,λ_p_ of the covariance matrix C are obtained using the following equation [71]:

(C‐λI)A=0 (13)

where I is the identity matrix and A is a matrix containing the set of eigenvectors of C corresponding to the set of eigenvalues λ. This equation has a nontrivial solution if and only if the matrix C−λI is not invertible and this happen if and only if λ satisfies the characteristic equation [71]:

determinant(C‐λI)=0 (14)

By solving this characteristic equation for λ, the p eigenvalues are obtained. Then, the eigenvalues are arranged in descending order, so that λ_j_≥λ_j+1_.

**Step 3:** the p eigenvectors of the covariance matrix C are calculated by taking each of its p eigenvalues (λ_1_, λ_2_,…,λ_p_) in turn, i.e., each eigenvector a_j_ corresponding to the eigenvalue λ_j_ where obtained by solving Eq (13) for λ = λ_j_, j = 1,2,…, p. Then A is a matrix whose columns are formed from eigenvectors of C, ordered whereas the first column of A is the eigenvector corresponding to the largest eigenvalue, and the last column is the eigenvector corresponding to the smallest eigenvalue. This will present the principle components in order of significance.

**Step 4:** finally, the p principal components are computed by Eq (11), each principal component is computed as Y_j_ = X a_j_.

Since the lower-dimensional representation has to be obtained from the largest eigenvalues only, that account for most of the variance, only their corresponding eigenvectors and principal components which must be used to describe the statistical features dataset. To do that, the percentage of the total variance of each principal component is computed by:

%oftotalvarianceofYj=λj∑j=1pλj (15)

where $\sum_{j=1}^{p}\lambda_{j}$ provides the total variance.

## Results and discussion

### Biospeckle image analysis

**Fig 4:**
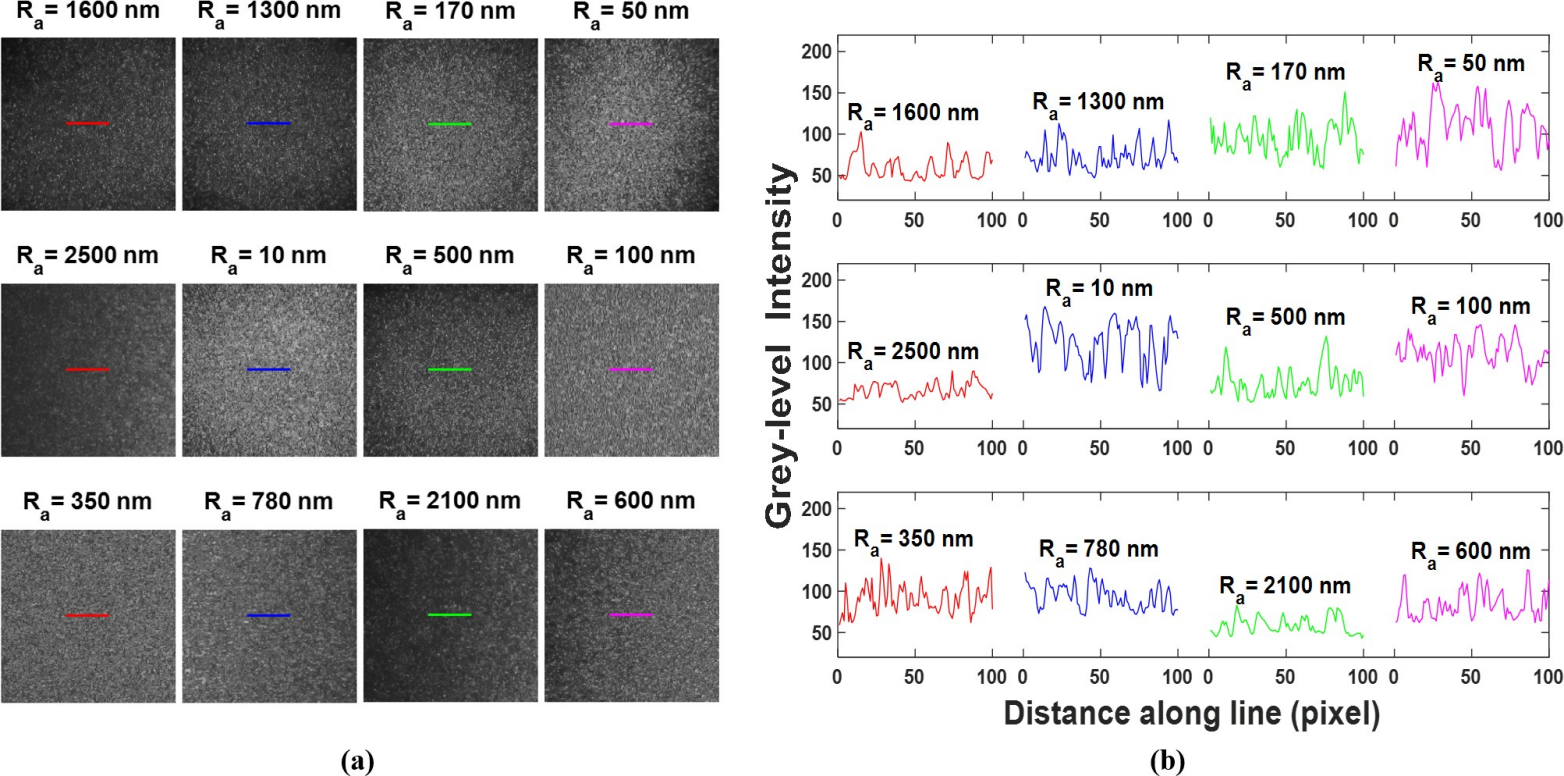
(a) Eight-bit gray-level display of twelve biospeckle images, with a dimension of 400×400 pixels, collected from twelve degenerated cross-section areas of a bovine articular cartilage specimen, and (b) gray-level intensity distribution along the colored lines passing over the biospeckle images presented in (a).

The biospeckle images presented in Fig 4(A) were collected using the experimental setup shown in Fig 3. The images were obtained from the backscattered radiations of twelve degenerated cross-section areas of the bovine articular cartilage tissue specimen having different average surface roughness values as discussed above. It can be observed that the biospeckle images contain mainly bright and dark batches with few gray-level values. In Fig 4(B), a gray-level intensity distribution along a line passing over each biospeckle image is presented. A closer look at the biospeckle images and line plots shows that the gray-level intensity decreases, the number of bright patches decreases, and the dark area increases as the average surface roughness increases. Besides, high gray-level transitions for the smooth surfaces than the rough ones are obvious in the line plots (Fig 4(B)), indicating that the rough surfaces have bright batches with a bigger size than the smooth surfaces. Clearly, the biospeckle images present texture patterns that differ for the different average surface roughness values. This outcome promoted the possibility to extract truthful statistical features from the biospeckle images to accurately estimate the surface topography.

### GLCM analysis

**Fig 5:**
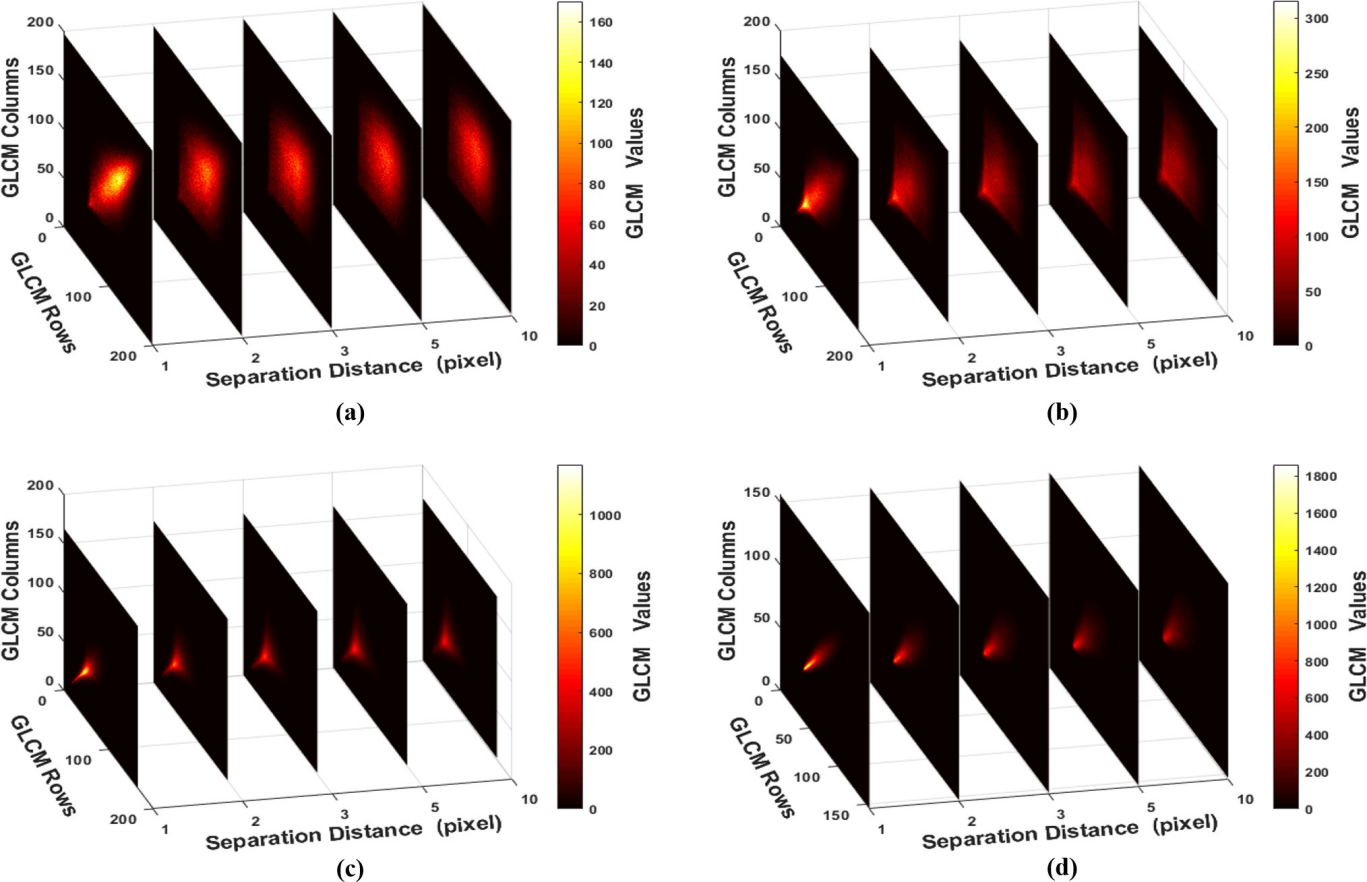
GLCM plots along the horizontal direction at d = 1,2,3,5 and 10 pixels of biospeckle images collected from cross-section areas of: (a) Ra = 10 nm, (b) Ra = 170 nm, (c) Ra = 1600 nm and (d) Ra = 2500 nm.

The GLCM was implemented from the relationship between two neighbor pixels at a certain separation distance along a specific direction. For each biospeckle image, forty GLCMs at ten separation distances (d = 1–10 pixels) along the four directions were implemented. Twenty of them concerning four different biospeckle images along the horizontal direction at five different separation distances are plotted and presented in Fig 5. It can be observed that as the average surface roughness increases, the element distribution width decreases where the elements concentrate towards the principal diagonal. This effect is due to that the biospeckle image of the rough surface has larger bright batches and less gray-level transitions than the smooth surface. Then, it implements GLCM with fewer entries with high values within the principal diagonal region unlike the other biospeckle image of the smooth surface which modifies the GLCM cells with more entries with low values away from the principal diagonal. Besides, the GLCM cell’s distributions moved towards the upper side direction of the matrix as the surface roughness increase (due to the lower gray-level intensities) resulting in higher values. What’s more, the cell’s distribution width increases with increasing the separation distance for each average surface roughness. The effectiveness of the GLCM in the discrimination of the biospeckle images is evident where the cells’ distributions of the GLCM differ in a good relationship with the separation distance and surface roughness. It is worthily to conclude that the GLCM contains important information about the gray-level intensities and spatial distribution of the biospeckle image pixels.

### Haralick’s statistical parameters extraction

**Fig 6:**
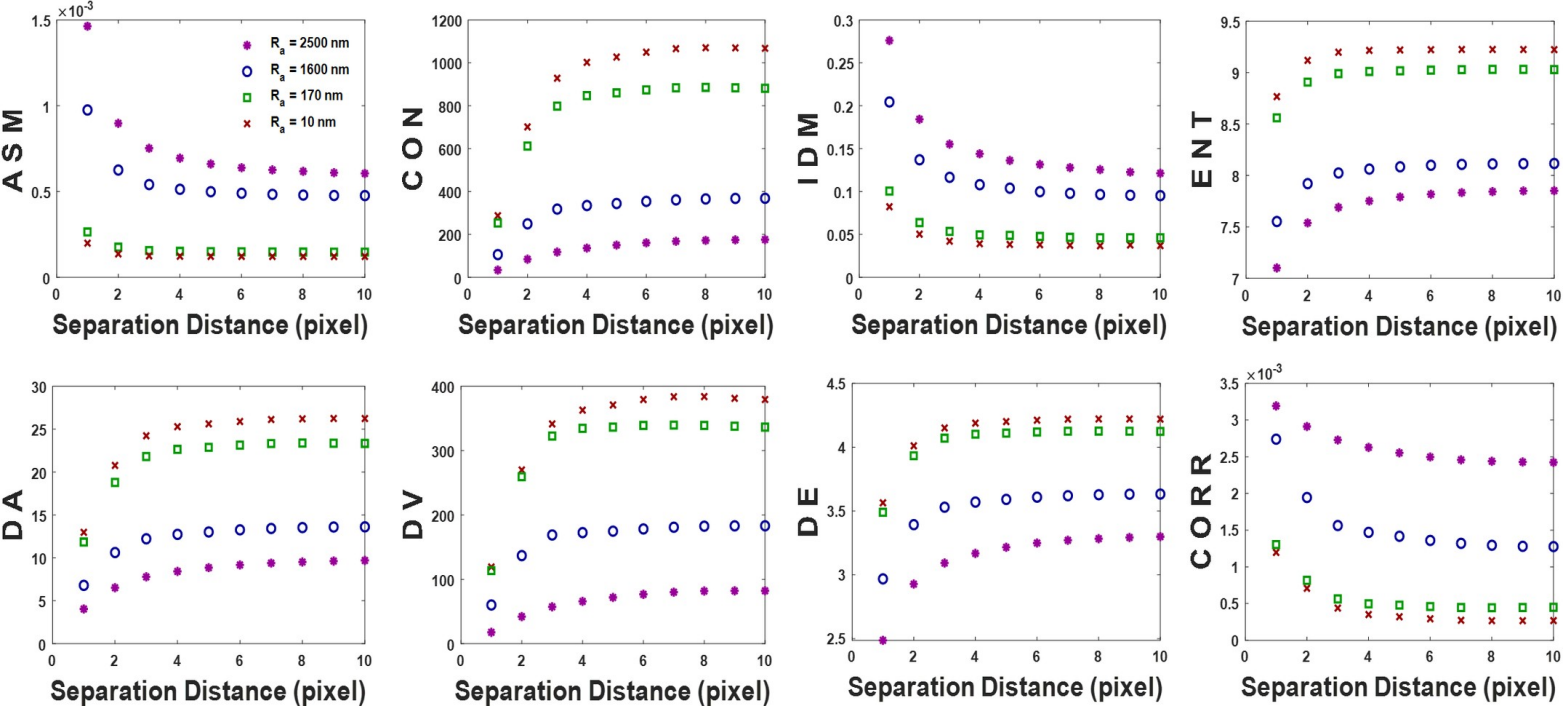
Plots of the extracted Haralick’s statistical parameters from the GLCM of four biospeckle images along 0° direction versus the separation distance.

From each implemented GLCM, the eight Haralick’s statistical parameters, angular second moment, contrast, inverse difference moment, entropy, difference average, difference variance, difference entropy, and correlation were computed. For illustrative purposes, Fig 6 shows the results of the extracted statistical parameters versus the separation distance of the biospeckle images whose GLCM plots are presented in Fig 5. Some explanations and discussions regarding both Figs 5 and 6: the ASM value outputted higher values with the increase in surface roughness and decrease in separation distance due to the increased sum of the squares of the P matrix values; that results from the domination of few numbers with large magnitude. Moreover, since the IDM and CORR Equations were created to measure the closeness of the cells’ distribution of the P matrix to the principal diagonal and cells’ correlations, respectively, they had high values for the biospeckle images with the big bright patches that were collected from rough surfaces. On the contrary, the CON, ENT, DA, DV, and DE outputted high values for the biospeckle image with rather small bright patches, as the GLCM is implemented from more entries with small values. Consequently, as the separation distance increased, the GLCM cells were distributed with small values away from the principal diagonal, hence high CON, ENT, DA, DV, and DE were obtained. Then, the extracted Haralick’s statistical parameters plots for the four cross-section areas seem to have good relationships with different ratings and dynamic range versus the separation distance and average surface roughness where they verify that the statistical parameters could describe the GLCM.

**Fig 7:**
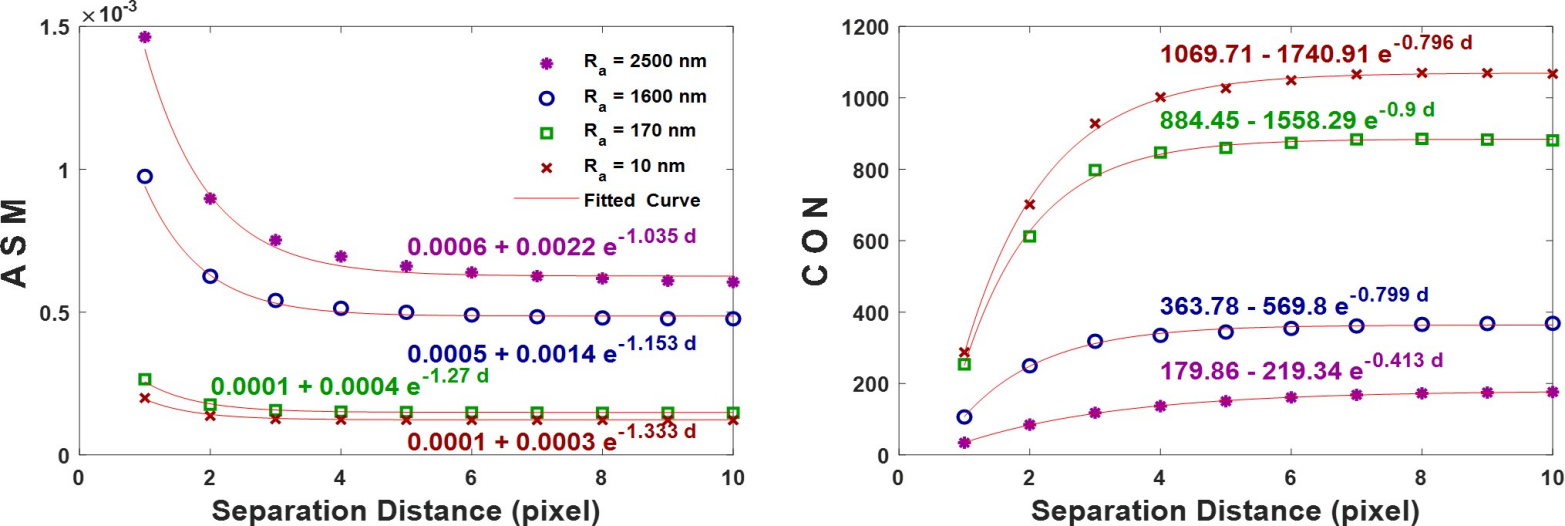
Exponential function fit for two of the extracted Haralick’s statistical parameters shown in Fig 6. The curve fitting equations are presented.

It was observed that all the extracted statistical parameters, presented in Fig 6, might resemble exponential function. As an empirical fitting model, the exponential function, y = *y*_0_+Ae^-Bx^, was utilized to fit the plots. Where y and x, respectively, indicate the statistical parameter value and the separation distance d, A and B are the exponential coefficients that together determine the shape and behavior of the exponential function (A is the y-intercept), and *y*_0_ is the constant that shifts the exponential curves vertically upwards or downwards. Examples of fitting exponential curves for two statistical parameters exhibiting exponential decay and growth, like ASM and CON respectively, are presented in Fig 7. Clearly, the fitting exponential function could accurately describe the different statistical parameters’ curves showing perfect fit correlation value, R^2^, of 0.99. As seen from the different fitting exponential curves, *y*_0_ has a good relationship with the average surface roughness. While A and B, which control the exponential function’s shape, are not promising to characterize the average surface roughness. Therefore, it was decided to ignore A and B and maintain *y*_0_ for each statistical parameter.

**Fig 8:**
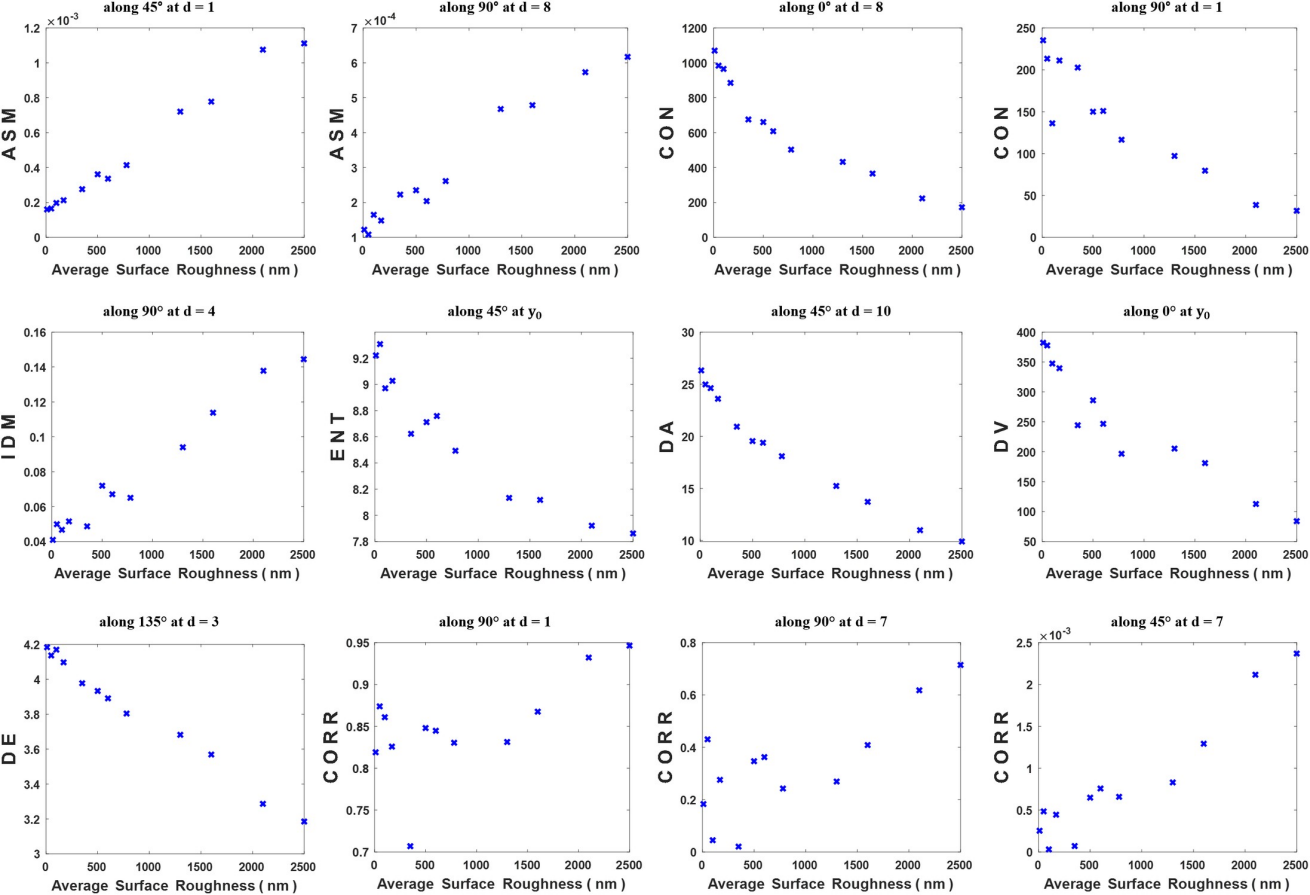
Computed Haralick’s statistical parameters versus the average surface roughness of the twelve articular cartilage cross-section areas.

To show the relationship between the statistical parameters and the average surface roughness for the twelve cross-section areas of the articular cartilage specimens, some plots chosen arbitrarily from the computed dataset of Haralick’s statistical parameters are presented in Fig 8. From the results of CORR (Fig 8(J)–8(L)), obtained along 90° and 45° where extremely different dynamic range is shown, it is interesting to note that the shape of the biospeckle image is not uniformly distributed in the four directions. Moreover, it is clearly observed that the statistical parameters have different distributions, rating, and dynamic range with respect to the average surface roughness and separation distance. Theses analysis reveals that each statistical parameter contains some specific information about the texture characteristics of the biospeckle image. Therefore, it is difficult to adopt a specific statistical feature, along a specific direction at a specific separation distance, to estimate the average surface roughness of the articular cartilage tissue specimens. On that point, it was suggested that the combination of these extracted Haralick’s statistical parameters together through the principal component analysis to produce new uncorrelated significant statistical features would add value in characterizing the average surface roughness.

### Dimensionality reduction by principal component analysis

**Fig 9:**
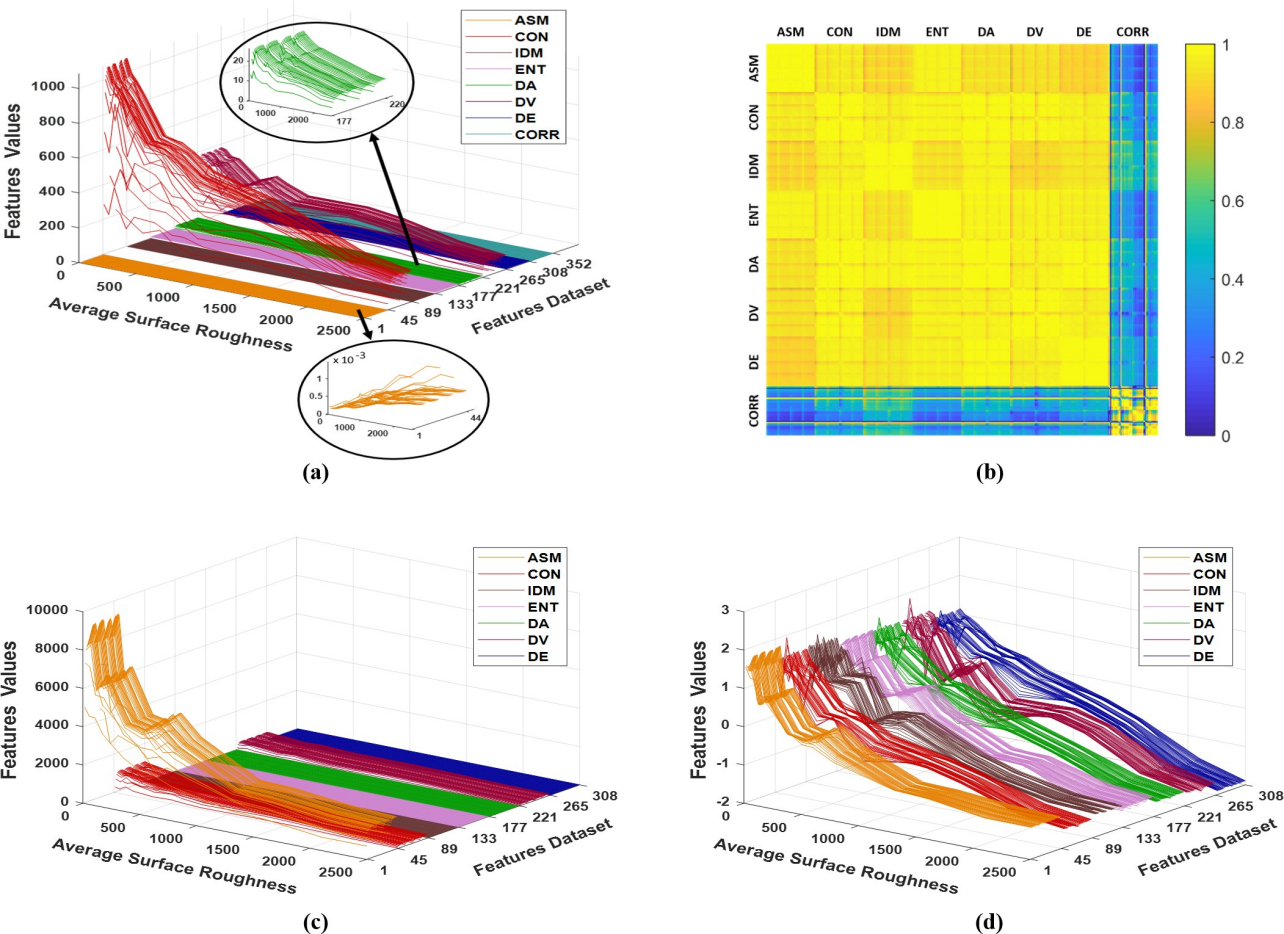
The distribution of the statistical features, 44 features for each Haralick’s statistical parameter, computed from the twelve cross-section areas of articular cartilage tissue: (a) the initial statistical features dataset, (b) the pairwise correlation matrix of the statistical features, (c) the statistical features dataset after having the same rating and removing the 44 statistical features of CORR, and (d) the final statistical features dataset after standardization to be analyzed by PCA, where they range from -1.92 to 2.09.

To prepare the statistical features dataset, each computed Haralick’s statistical parameter extracted from the GLCM at a specific direction and separation distance was used as a feature. Therefore, for each Haralick’s statistical parameter, 44 statistical features were obtained at d = 1–10 and their fitting constant *y*_0_, along the four directions. That is to say, the statistical feature dataset comprised p = 352 statistical features for each observation (i.e. cross-section area of the articular cartilage tissue specimens, n = 12). Summing up, the feature dataset, shown in Fig 9(A), is represented by 12×352 data matrix X whose j^th^ column is a vector x_1:12,j_ of observations described by the j^th^ statistical feature.

Before analyzing the statistical features dataset using PCA: first and most importantly, all the computed features of ASM, IDM, and CORR (along all directions and separation distances, and their fitting parameter y_0_) were replaced by their reciprocal (multiplicative inverse). Therefore, all the statistical features could have the same rating, i.e. as the average surface roughness increase, they decrease (see Fig 9(A) and 9(C)). Second, the strength and direction of the relationship between the 352 statistical features, were measured through computing the pairwise correlation among them by Eq (5) and presented in Fig 9(B). The principal diagonal of the correlation matrix represents the highest correlation value since the pairwise correlation is computed between a feature and itself. It is apparent from this figure that there are significantly high pairwise correlation coefficients among the statistical features (more than 0.80) except for the 44 statistical features of CORR which show very weak correlation values with the other statistical features. The presumed reason is the nonuniform distribution of the biospeckle image in different directions, resulting in inconsistent CORR values for describing the average surface roughness. As a result, the 44 statistical features of CORR were excluded from the statistical features dataset. Then the dataset X became a matrix of order 12×308, where p = 308. Third, since the extracted Haralick’s statistical parameters had different dynamic ranges as evident in Figs 8 and 9(C), the statistical feature data were standardized to have the same dynamic range and weight by means of Eq (10). Fig 9(C) and 9(D) show the distribution of the statistical features data before and after standardization, respectively.

**Fig 10:**
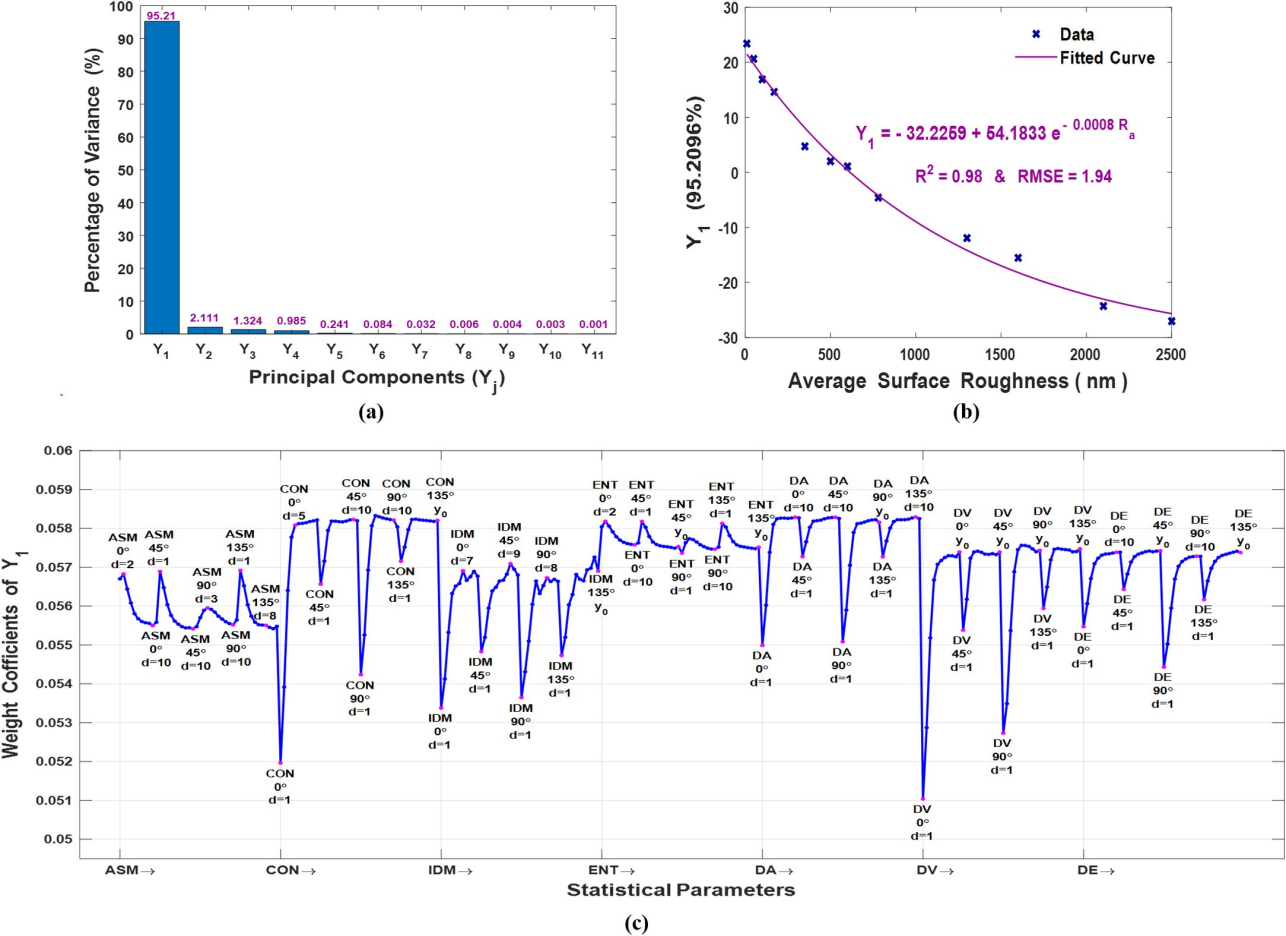
Principal component analysis: (a) percentage of the total variance of the principal components, (b) the first principal component versus average surface roughness, and (c) the weight coefficients plot of the first principal component.

After preparing the statistical features dataset to be analyzed by PCA, the eigenvalues, p = 308, and their corresponding eigenvectors, A = 308×308, were obtained. Then, utilizing Eq (11), a new statistical feature vector without redundant information, a set of principal components that do not exhibit correlation among them ordered according to their meaningful (from the highest to the lowest eigenvalues), were generated. The dimensionality of the statistical features’ dataset was effectively reduced to a feature vector of only eleven principal components. The percentage of the total variance explained by the feature vector of the principal components is shown in Fig 10(A), in which it is clearly seen that the first principal component by itself explains more than 95% of the total variance. Therefore, more components were not needed as they were less significant, the second and third principal components explain 2.111% and 1.324% of the total variance, respectively. It must be declared that these percentages are consequent to the high correlation among the statistical features. The relationship between the first principal component and the average surface roughness of the twelve articular cartilage cross-section areas is plotted in Fig 10(B). As shown in the plot, the fitting curve of the data can resemble an exponential function with fit correlation and root-mean-square error of R2 = 0.98 and RMSE = 1.94, respectively. Since the first principal component eigenvector which consists of 308 weight coefficients indicates how each statistical feature contributes to it, its plot is illustrated in Fig 10(C). It has been found that the first principal component has positive weight coefficients for all the statistical features. Whereas the largest coefficients are corresponding to the statistical features of CON and DA at d ~ 5–10 pixels and y_0_. While the smallest coefficients are corresponding to DV along 0° and 90° at d = 1, and CON along 0° at d = 1. The obtained result reveals that the first principal component can accurately estimate the average surface roughness of the articular cartilage tissue surface.

## Conclusion

Investigating a new diagnostic non-contact and non-destructive method that has the ability to quantitively measure the nanoscale surface roughness of articular cartilage tissue, which is the earliest important indicator of osteoarthritis, has been proposed. The method has been based on second-order statistical-based biospeckle optical imaging through the combination of highly correlated statistical features extracted from the gray-level co-occurrence matrix by means of principal component analysis. The results showed that the first principal component which explains more than 95% of the total variance has positive weight coefficients for all the statistical features and can accurately discriminate the different degenerated cross-section areas of the articular cartilage tissue. Therefore it was turned out to be a significant and valuable statistical feature. In conclusion, the proposed method can be used as an alternative tool for early diagnosis of articular cartilage degeneration and it is expected that the proposed statistical analysis of biospeckle images will be widely used by researchers for precise analysis of biospeckle images in different applications.
